# A longitudinal dyadic analysis of financial strain and mental distress among different-sex couples: the role of gender division of labor in income and housework

**DOI:** 10.1186/s12889-025-22059-x

**Published:** 2025-03-05

**Authors:** Sizhan Cui, Fenwick Feng Jing, Haoyuan Ma, Meng Zhu, Yaguang Yan, Senhu Wang

**Affiliations:** 1 Nanjing University, Nanjing, China; 2 Qufu Normal University, Qufu, China; 3 Nanjing Foreign Language School, Nanjing, China; 4 Hubei University of Economics, Wuhan, China; 5 Nippon Bunri University, Oita, Japan; 6 National University of Singapore, Singapore, Singapore; 7 University of Cambridge, Cambridge, UK

**Keywords:** Financial strain, Mental distress, Gender, Couples, Division of labor

## Abstract

**Background:**

Despite extensive research on the association between financial strain and mental distress, little is known about its spousal crossover effect in different-sex couples.

**Methods:**

Using nationally representative longitudinal dyadic data from the UK and fixed effect models, this study examines the dyadic relationship between financial strain and mental distress among couples, and the different relationship by gendered division of labor in income and housework.

**Results:**

Our findings reveal that for both the husband and wife, financial strain is not only associated with their own mental distress but also their spouses’ mental distress, although the spousal cross-over effects have smaller effect sizes. Next, the husband’s mental distress associated with his own financial strain decreases as his share of the couple’s income increases, while his income share does not significantly alleviate his wife’s mental distress related to her own or her husband’s financial strain. Lastly, the wife’s share of housework does not modify the effect of financial strain on her husband’s or her own mental distress. These findings suggest the protective role of the traditional breadwinner in alleviating the husband’s mental distress from his own financial strain, but not from his wife’s financial strain, while also highlighting the limited effects of housework in mitigating the psychological toll of financial strain on both partners.

**Conclusions:**

Overall, these findings provide valuable insights into our understanding of the relationship financial strain and mental distress by incorporating the dyadic perspective and gendered division of labor.

## Introduction

In the contemporary society, increasing financial strain is becoming a prominent concern due to labor market uncertainty, rising unemployment rates, and precarious employment conditions. These economic challenges can significantly contribute to mental distress, manifesting as anxiety, depression, and other stress-related disorders among influenced individuals [1, 2]. Studying the effects of financial strain on mental distress not only allows for the identification of those most vulnerable to the psychological effects of economic instability, but also can guide the development of comprehensive social and economic policies aimed at alleviating both the financial and psychological burdens faced by individuals [3, 4]. So far, existing research has consistently showed that financial strain is significantly related to higher risks of developing depression, anxiety and other related mental health problems [5–7]. This line of research underscores the intricate link between economic well-being and mental health, advocating for integrated approaches that consider financial stability as a fundamental aspect of promoting overall mental wellness and preventing mental health disorders.

However, these studies often overlook the interconnected nature of couples’ lives, especially in different-sex relationships, where societal norms and gender roles can have effects on how stress is experienced and managed [1, 8]. Hence, this study uses nationally representative longitudinal dyadic data from the UK and fixed effect models to achieve two objectives. First, we attempt to discern the effects of financial strain on mental distress for both husbands and wives among married or co-residing couples.^1^ Second, we aim to examine whether the effects of financial strain on mental distress depends on gender role divisions in income and housework in order to uncover how traditional and evolving gender norms shape the psychological resilience or vulnerability of individuals within intimate partnerships.

Drawing on the “linked lives” perspective, we expect that financial strain not only influences one’s own mental health, but also has cross-over effect on his or her spouse’s health and wellbeing. The spousal crossover effect is encapsulated by the “linked lives” thesis, which highlights the profound effect that one spouse’s characteristics can have on the other’s well-being [1, 9]. This perspective highlights the importance of considering both partners in a relationship when addressing mental health issues, recognizing that financial strain can create a ripple effect that extends beyond the individual to influence the well-being of the couple as a whole [10].

### Financial strain and mental distress

In the context of financial strain, several mechanisms explain how spousal economic hardship influences personal well-being. First, the crossover effect shows that one partner’s financial strain can lead to increased mental distress in the other partner, revealing a shared vulnerability to economic pressures [6, 11, 12]. Second, financial strain from unemployment and job insecurity can deteriorate relationship quality, resulting in more arguments and less emotional support between partners due to a shared sense of instability and anxiety [13]. Understanding this spousal crossover effect is crucial, as it can reveal broader patterns of financial wellbeing and mental health within households, offering insights into how policies might be tailored to better support not just individuals, but families as a whole [5, 7]. Thus, this study aims to advance and contribute to the existing literature by transcending the traditional individual-level examination of financial wellbeing and health, to explore the spousal crossover effect within couples. Based on the above discussion, we propose the following hypotheses.

#### *H1*

*Husband’s financial strain has positive effects on his own and his wife’s mental distress.* 2

#### *H2*

*Wife’s financial strain has positive effects on her own and her husband’s mental distress.*

### The role of division in income

Next, regarding the potential effect of the husband’s and wife’s financial strain depending on gender role division in income, we outline two different expectations for the husband’s mental distress.

First, according to traditional gender roles, where the husband is the primary breadwinner and the wife manages household tasks, these roles buffer the husband from both his own and his wife’s financial hardships [14]. This arrangement aligns with societal expectations and, in turn, may bolster the husband’s self-esteem and sense of control over his life [15, 16]. Additionally, the psychological benefits of being the primary breadwinner include feelings of pride and accomplishment, which can counterbalance the negative emotions associated with financial struggles within the household [17]. This is because the husband’s larger income share not only stabilizes the household’s finances but also reinforces his role within the family, helping to mitigate the indirect effects of financial strain on his mental well-being [18–20]. Conversely, the effects of both the husband’s and the wife’s financial strain on his mental distress can be intensified when the husband has a smaller share of the couple’s income. This occurs because a diminished financial contribution may lead to feelings of inadequacy or loss of identity, especially when traditional gender norms emphasize the male breadwinner role [18, 21].

Second, with the rise of dual-earner households in today’s labor market [22], relying on both partners’ financial resources can mitigate the effects of the husband’s and wife’s financial strain on his mental distress [23]. This happens because an equal share of income reduces the husband’s financial pressure [24, 25]. However, the effects of the husband’s and wife’s financial strain on his mental distress can intensify when the income share is unequal, manifesting in two scenarios. When the husband is the primary breadwinner, the effects of both his own and his wife’s financial strain on his mental distress can be more pronounced. This is a risky strategy, especially if he loses his job or faces other financial setbacks [26]. Conversely, when the wife is the primary breadwinner, the effects of the husband’s and wife’s financial strain on his mental distress can also increase. This occurs because it violates traditional gender norms, leading to additional psychological stress [21, 27].

Thus, we propose the following hypotheses.

#### *H3a*

*For the husband’s mental distress*, *the positive effects of both his own and his wife’s financial strain are less pronounced when he has a larger share of the couple’s income.*

#### *H3b*

*For the husband’s mental distress*, *the positive effects of both his own and his wife’s financial strain are less pronounced when the couple has a more equal share of income compared to when the husband earns more or less than his wife.*

Regarding the potential effect of the husband’s and wife’s financial strain depending on gender role division in income, we also outline two different expectations for the wife’s mental distress.

First, despite the increasing workforce participation of women in the UK, traditional gender roles that position men as breadwinners and women as homemakers remain influential [28, 29]. Consequently, we expect that the positive effects of both the wife’s own and her husband’s financial strain on her mental distress are less pronounced when the husband has a larger share of the couple’s income. This occurs because the husband’s financial strain may place less pressure on the wife to contribute to the household’s financial resources, potentially reducing her exposure to financial stress [14, 17]. Furthermore, the wife’s mental health is also less adversely influenced by her husband’s financial strain when the family income predominantly relies on the husband [30, 31]. This is because the perceived relative financial stability and the husband’s fulfillment of his breadwinner role within the household serve to cushion the psychological effect of financial challenges [32–34]. However, the effects of the husband’s and wife’s financial strain on the wife’s mental distress can be intensified when the husband has a smaller share of the couple’s income. This is due to the wife potentially feeling an increased burden to compensate for the husband’s lower financial contribution, which leads to heightened stress and anxiety as she struggles to meet the household’s financial needs [26, 35].

Second, we expect that the positive effects of both the wife’s own and her husband’s financial strain on her mental distress remain consistent regardless of her husband’s share of income. This is because, as women increasingly become economically independent, their mental health is often more closely tied to their own economic conditions and role-related stress than to their husband’s income share [30, 36]. Specifically, the effects of the husband’s and wife’s financial strain on her mental distress remain unchanged when her husband’s income share fluctuates. When the husband contributes a larger share of the income, the wife may still experience consistent mental distress if her own or her husband’s financial strain persists. This occurs because the wife might feel a sense of responsibility to alleviate the financial pressure on her husband, leading to anxiety and guilt if she feels unable to contribute more significantly [37, 38]. Conversely, when the husband contributes a smaller share of the income, the wife may still experience consistent mental distress if her own or her husband’s financial strain persists. This happens because the wife may feel an overwhelming pressure to be the primary financial provider, resulting in anxiety and burnout if her efforts are insufficient to meet the household’s needs [39, 40].

Thus, we propose the following hypotheses.

#### H4a

*For the wife’s mental distress*, *the positive effects of both her own and her husband’s financial strain are less pronounced when the husband has a larger share of couple’s income.*

#### H4b

*For the wife’s mental distress*, *the positive effects of both her own and her husband’s financial strain remain consistent regardless of her husband’s share of income.*

### The role of division in housework

Lastly, concerning whether and how the effects of the husband’s and wife’s financial strain on mental distress depend on gendered divisions in housework, we outline two distinct expectations for the husband’s mental distress.

First, according to classical gender theories, the husband tends to prioritize financial and economic responsibilities over household duties, which means his mental distress is not influenced by the division of housework [41–43]. This is partly because the husband is typically less involved in household duties [27, 38]. Specifically, the effects of the husband’s and wife’s financial strain on his mental distress can remain unchanged when the wife’s share of housework changes, encompassing two scenarios. When the wife contributes a larger share of housework, the husband may still experience consistent mental distress if his own or his wife’s financial strain persists. This is because persistent financial challenges can overshadow household management benefits, as the husband’s focus on financial pressures keeps his mental distress unchanged [44, 45]. Conversely, when the wife contributes a smaller share of housework, the husband may still experience consistent mental distress if his own or his wife’s financial strain persists. This is due to potential frustration over the imbalance in contributions, combined with his reluctance to do housework, which he views as unpleasant and typically only takes on when he has less power in the relationship [46].

Second, according to expansionist theory [37], an equal share of housework can mitigate the effects of the husband’s and wife’s financial strain on his mental distress. This occurs because it reduces the pressure on him to be the primary provider, as he values success through both financial contributions and active participation in household duties [38, 47]. However, the effects of the husband’s and wife’s financial strain on his mental distress can be intensified when the housework share is unequal, encompassing two scenarios. When the husband assumes the primary role in housework, the effects of the husband’s and wife’s financial strain on his mental distress can be more pronounced. This occurs because taking on the primary role in housework may conflict with traditional gender norms, leading the husband to feel inadequate or emasculated [27]. Conversely, when the wife assumes the primary role in housework, the effects of the husband’s and wife’s financial strain on his mental distress can also be more pronounced. This is because the husband might feel additional pressure to compensate for the financial strain due to traditional masculine norms, which can heighten his stress and sense of inadequacy [18, 48].

Thus, we propose the following hypotheses.

#### *H5a*

*For the husband’s mental distress*, *the positive effects of both his own and his wife’s financial strain remain consistent regardless of his wife’s share of housework.*

#### *H5b*

*For the husband’s mental distress*, *the positive effects of both his own and his wife’s financial strain are less pronounced when the couple has a more equal share of housework compared to when the husband does less or more housework than his wife.*

For the wife, the effects of her own or her husband’s financial strain on her mental distress are expected to be influenced differently by her share of housework. We outline two different expectations for this relationship.

First, according to the gender ideology or doing-gender perspective, the positive effects of both her own and her husband’s financial strain remain consistent irrespective of her share of housework. This is because financial strain and the resulting stress for the wife are intrinsically linked to economic roles and responsibilities rather than to household labor divisions [49, 50]. Specifically, the effects of both partners’ financial strain on her mental distress can remain unchanged regardless of variations in her share of housework. This consistency can be observed in two scenarios. When the wife undertakes a larger share of housework, she may still experience consistent mental distress if either her own or her husband’s financial strain persists. This is because she feels that her increased efforts in housework are insufficient to mitigate financial pressure [51]. Conversely, when the wife undertakes a smaller share of housework, she may still experience consistent mental distress if either her own or her husband’s financial strain continues. This is because she perceives that her reduced share of housework does not offset the financial strain being experienced [38].

Second, according to the theory of equity, an equal distribution of housework can mitigate the effects of both the husband’s and wife’s financial strain on the wife’s mental distress [52]. This balance can improve relationship satisfaction and communication, creating a more supportive environment that enables the wife to better manage financial stress [53, 54]. However, the effects of the husband’s and wife’s financial strain on her mental distress may be exacerbated when the distribution of housework is unequal, which can manifest in two ways. When the wife is primarily responsible for housework, the effect of both the husband’s and wife’s financial strain on her mental distress may be more pronounced. This is because the wife has to shoulder the dual burden of managing most household responsibilities while coping with financial difficulties [55]. Conversely, when the husband assumes primary responsibility for housework, the effects of the husband’s and wife’s financial strain on the wife’s mental distress may also be more significant. This might challenge traditional gender norms and lead to the wife’s feelings of guilt or inadequacy, potentially resulting in additional stress [27, 56].

Thus, we propose the following hypotheses.

#### *H6a*

*For the wife’s mental distress*, *the positive effects of both her own and her husband’s financial strain remain consistent regardless of her share of housework.*

#### *H6b*

*For the wife’s mental distress*, *the positive effects of both her own and her husband’s financial strain are less pronounced when the couple has a more equal share of housework.*

## Methods

### Data and sample

The data used in this study come from the first (2009–2011), second (2010–2012), fourth (2012–2014), sixth (2014–2016), eighth (2016–2018), tenth (2018–2020) and twelfth (2020–2022) waves of Understanding Society: The United Kingdom Household Longitudinal Study (UKHLS), which is one of the biggest and most widely used household panel survey datasets in the UK [57]. Only these seven waves of the UKHLS cover all the key variables we need. The UKHLS collected a stratified clustered sample of around 30,000 UK households in 2009, which builds on and incorporates its predecessor, the British Household Panel Survey (1991–2008). It gathers extensive data on socio-economic characteristics, employment status, health, and well-being. The University of Essex Ethics Committee has approved all data collection. To construct our analytic sample, we restricted the sample to heterosexual married or cohabiting couples who completed the self-completion questionnaire. After listwise-deleting a small number of observations with missing values (4.15% of the sample), the final analytic sample contains 59,753 couple–years (16,171 couples).

### Measures

The dependent variable is mental distress, which is measured by the 12-item of General Health Questionnaire (GHQ), a consistent and reliable indicator of psychiatric illness and distress. The GHQ-12 asked respondents 12 questions about their depressive and anxiety symptoms, sleeping problems as well as overall happiness etc. (for more details, see understandingsociety.ac.uk). UKHLS converts the answers to GHQ-12 questions to a single continuous scale ranging from 0 (the least distressed) to 36 (the most distressed) [58].

Our key independent variable is financial strain. To measure this variable, respondents were asked to rate their current financial situation on a five-point scale ranging from 1 (living comfortably) to 5 (finding it very difficult) [2].

Our primary moderating variables are the gendered division of labor in income and housework, which we measure by the husband’s share of the couple’s income and the wife’s share of total housework time per week. The income of both husbands and wives is calculated as the total monthly net income, including labor income, miscellaneous income, private benefit income, investment income, pension income, and social benefit income. The total housework time per week for husbands and wives is measured by the hours they spend on household tasks each week. To mitigate the effects of potential outliers and ensure the robustness of our measures, income and housework time have been top-coded at the 99th percentile prior to calculating the percentages.

In addition, we controlled for a number of socio-demographic variables. Specifically, we adjusted for individual features including age (continuous), employment status (no paid work, self-employment, paid employment), and education level (below tertiary, tertiary). In addition, we also considered several couple- or household-level characteristics such as the couple’s marital status (unmarried cohabitation, married), parenthood (no dependent children, dependent children), and equivalised household income (log) (continuous, top-coded at the 99th percentile). Finally, we also included survey wave dummies and UK nations (England, Wales, Scotland, Northern Ireland) to adjust for the period and regional effects.

### Analytic strategies

All statistical analysis, tables, and figures were performed using Stata 17.

First, in descriptive analysis, all the analytical variables were examined using T-test and chi-squared tests to examine whether husbands and wives differ in mental health, subjective financial situation, and sociodemographic characteristics.

Second, fixed effect models with clustered standard errors were used to account for the interpersonal effects and dyadic interdependence between spouses, and to examine the differences in mental health effects of the financial situation between husbands and wives while controlling for sociodemographic variables. To ensure robustness, we have also implemented the APIM by estimating generalized structural equation models (GSEM) with random intercepts and clustered standard errors at the couple level (in Appendix Table A3, Table A4, and Figure A1), accounting for the panel data structure (i.e., couple-wave observations nested within couples). The results are consistent with those from the fixed-effect models, confirming the robustness of our findings.

## Results

### Descriptive statistics

The characteristics of the sample are detailed in Table 1. Overall, wives reported statistically higher mental distress and lower perceived financial strain compared to husbands (*p* < 0.001).

**Table 1:** Sample characteristics

Variables	Husband	Wife	Min	Max	*T/χ* ^*2*^ *tests*
Mental distress, *M* (*SD*)	10.33 (4.84)	11.31 (5.32)	0	36	*p* < 0.001
Perceived financial strain, *M* (*SD*)	2.03 (0.96)	2.01 (0.95)	1	5	*p* < 0.001
Age, *M* (*SD*)	52.92 (15.18)	50.54 (15.01)	16	99	*p* < 0.001
Marital status ^b^, %					
Unmarried cohabitation	15.66	—			
Married	84.34	—			
Parenthood ^b^, %					
No dependent children	60.13	—			
Have dependent children	39.87	—			
Equivalised household income (log) ^ab^, *M (SD)*	7.54 (0.68)	—	0	9.88	
Husband’s share of income ^ab^, *M (SD)*	0.59 (0.17)	—	0.07	1	
Wife’s share of housework ^ab^, *M (SD)*	0.66 (0.21)	—	0.02	0.99	
Employment status, %					*p* < 0.001
No paid work	34.13	43.29			
Self-employment	12.78	5.83			
Paid employment	53.08	50.88			
Education level, %					*p* < 0.001
Below tertiary	59.38	56.90			
Tertiary	40.62	43.10			
UK Nations ^b^, %					
England	78.83	—			
Wales	6.87	—			
Scotland	8.82	—			
Northern Ireland	5.48	—			
Number of observations	59,753 couple–years (16,171 couples)				

*Notes* M = Means, SD = Standard deviations, % = Proportions

^a^ Top-coded at the 99th percentiles to reduce the influence of outlier cases

^b^ Couple-level variable

There were statistically significant differences in age between husbands and wives, with husbands being, on average, older than wives. The majority of respondents were married (84.34%), from England (78.83%), and did not have dependent children (60.13%). Additionally, there were statistically significant differences in employment status and education level between husbands and wives, with husbands being more likely to be in paid employment and less likely to hold a tertiary educational degree (*p* < 0.001).

### Fixed effect models

Results of fixed effect models examining the effects of the husband’s and wife’s financial strain on their own and their partner’s mental distress are presented in Table 2.

**Table 2:** Fixed effect models examining the relationships between financial strain and mental distress

	Husband’s mental distress	Wife’s mental distress
Husband’s financial strain	0.855^***^	0.200^***^
	(0.036)	(0.036)
Wife’s financial strain	0.140^***^	0.732^***^
	(0.033)	(0.040)
Constant	14.046^***^	15.158^***^
	(3.340)	(3.528)
Within R-squared	0.0422	0.0349
Number of couples	16,171	16,171
Number of couple-wave observations	59,753	59,753

Notes^*^p < 0.05, ^**^p < 0.01, ^***^p < 0.001; Clustered standard errors in parentheses; All models control for husband’s share of couple’s income, wife’s share of housework, husband’s and wife’s age, educational level, employment status, couple’s marital status, parenthood, equivalised household income (log), and UK nations

Our results reveal that both the husband’s and wife’s financial strain have a statistically significant and positive effect on the husband’s mental distress (B = 0.855, *p* < 0.001; B = 0.140, *p* < 0.001). Similarly, both the husband’s and wife’s financial strain statistically increase the wife’s mental distress (B = 0.200, *p* < 0.001; B = 0.732, *p* < 0.001). These results provide strong support to both H1 and H2.

### Interaction effects of gender division

Table 3 reports the interaction effects between financial strain and the gendered division of labor in income and housework on mental distress.

**Table 3:** Fixed effect regression models examining the interaction between financial strain, husband’s share of income and wife’s share of housework on mental distress

	Husband’s mental distress	Wife’s mental distress
**Panel A. Interaction between financial strain and husband’s share of income**		
Husband’s financial strain	1.771^***^	0.303
	(0.257)	(0.225)
Wife’s financial strain	0.275	0.931^***^
	(0.254)	(0.264)
Husband’s share of income	3.994^*^	2.132
	(1.712)	(1.839)
Husband’s share of income^2^	-1.652	-2.096
	(1.472)	(1.599)
Husband’s financial strain × Husband’s share of income	-2.452^**^	-0.167
	(0.910)	(0.841)
Wife’s financial strain × Husband’s share of income	-0.528	-1.001
	(0.889)	(0.966)
Husband’s financial strain × Husband’s share of income^2^	1.354	-0.017
	(0.791)	(0.766)
Wife’s financial strain × Husband’s share of income^2^	0.476	1.034
	(0.768)	(0.868)
Constant	11.166^**^	13.578^***^
	(3.397)	(3.542)
Within R-squared	0.0432	0.0340
Number of couples	16,171	16,171
Number of couple-wave observations	59,753	59,753
**Panel B. Interaction between financial strain and wife’s share of housework**		
Husband’s financial strain	1.293^***^	0.045
	(0.207)	(0.224)
Wife’s financial strain	0.115	0.680^**^
	(0.199)	(0.242)
Wife’s share of housework	-0.456	-4.320^**^
	(1.225)	(1.557)
Wife’s share of housework^2^	1.175	2.833^*^
	(0.962)	(1.176)
Husband’s financial strain × Wife’s share of housework	-1.040	0.622
	(0.679)	(0.735)
Wife’s financial strain × Wife’s share of housework	0.102	-0.019
	(0.659)	(0.788)
Husband’s financial strain × Wife’s share of housework^2^	0.523	-0.526
	(0.537)	(0.572)
Wife’s financial strain × Wife’s share of housework^2^	-0.082	0.128
	(0.523)	(0.612)
Constant	12.323^***^	15.597^***^
	(3.327)	(3.545)
Within R-squared	0.0421	0.0350
Number of couples	16,171	16,171
Number of couple-wave observations	59,753	59,753

Notes^*^p < 0.05, ^**^p < 0.01, ^***^p < 0.001; Clustered standard errors in parentheses; All models control for husband’s and wife’s age, educational level, employment status, couple’s marital status, parenthood, equivalised household income (log), and UK nations

Panel A examines the interaction effects between financial strain and husband’s income share on mental distress. We observe a statistically negative interaction between the husband’s financial strain and his income share on his own mental distress (B = -2.452, *p* < 0.01). This suggests that the adverse effect of financial strain decreases as the husband’s income share increases, though this relationship appears to be linear, as indicated by the statistically non-significant quadratic term (B = 1.354, *p* > 0.05). On the other hand, the interaction between the wife’s financial strain and husband’s income share on the husband’s mental distress is not statistically significant (B = -0.528, *p* > 0.05 for the linear term; B = 0.476, *p* > 0.05 for the quadratic term). Regarding the wife’s mental distress, neither the interaction between her financial strain and her husband’s income share (B = -1.001, *p* > 0.05 for the linear term; B = 1.034, *p* > 0.05 for the quadratic term) nor the interaction between her husband’s financial strain and her husband’s income share (B = -0.167, *p* > 0.05 for the linear term; B = -0.017, *p* > 0.05 for the quadratic term) is statistically significant. This suggests that the buffering effect of being the primary breadwinner is more evident for the husband’s mental distress. These results lend support to H3a and H4b.

**Fig. 1:**
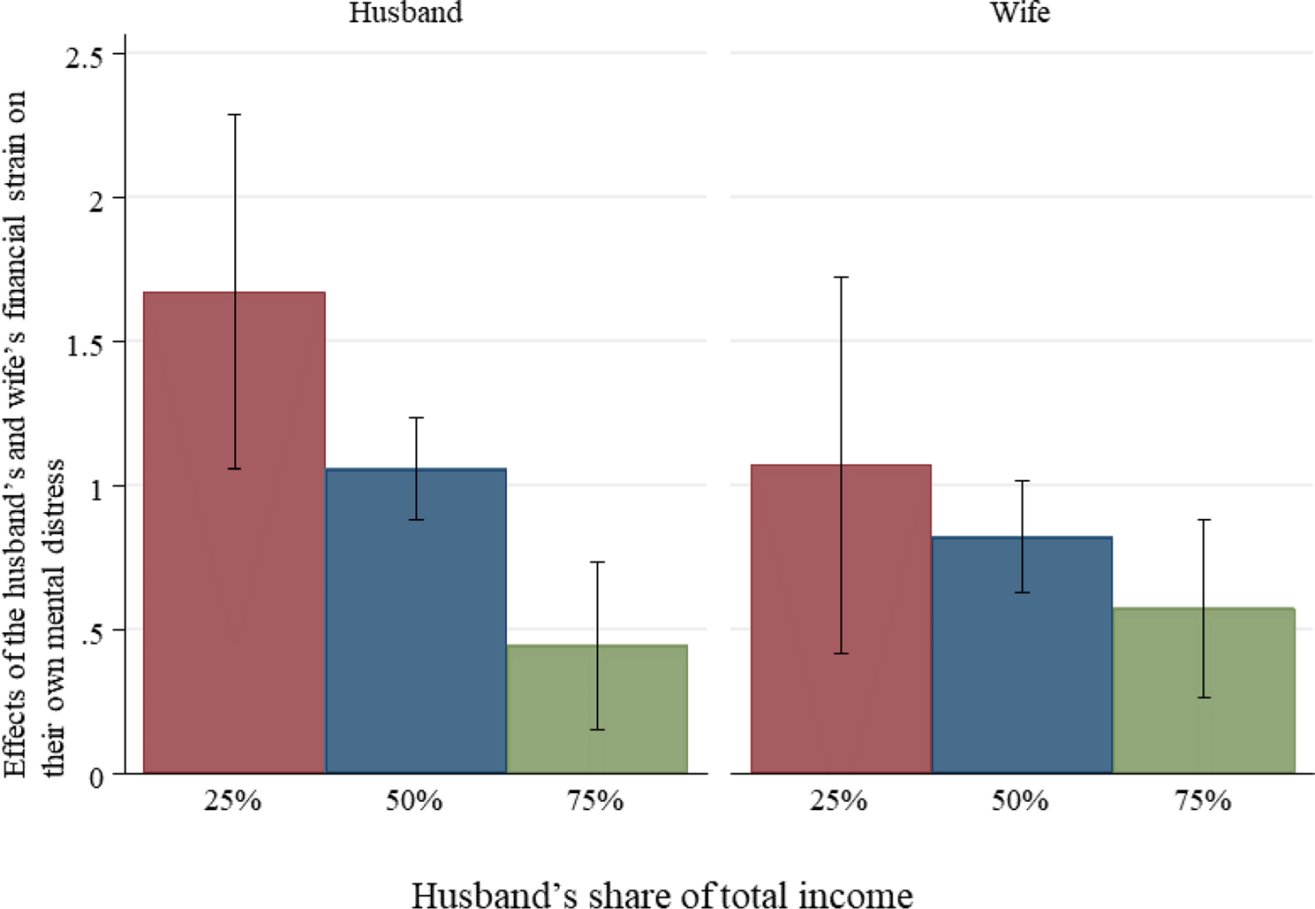
Effects of the husband’s and wife’s financial strain on their own mental distress by the husband’s share of couple’s income

**Fig. 2:**
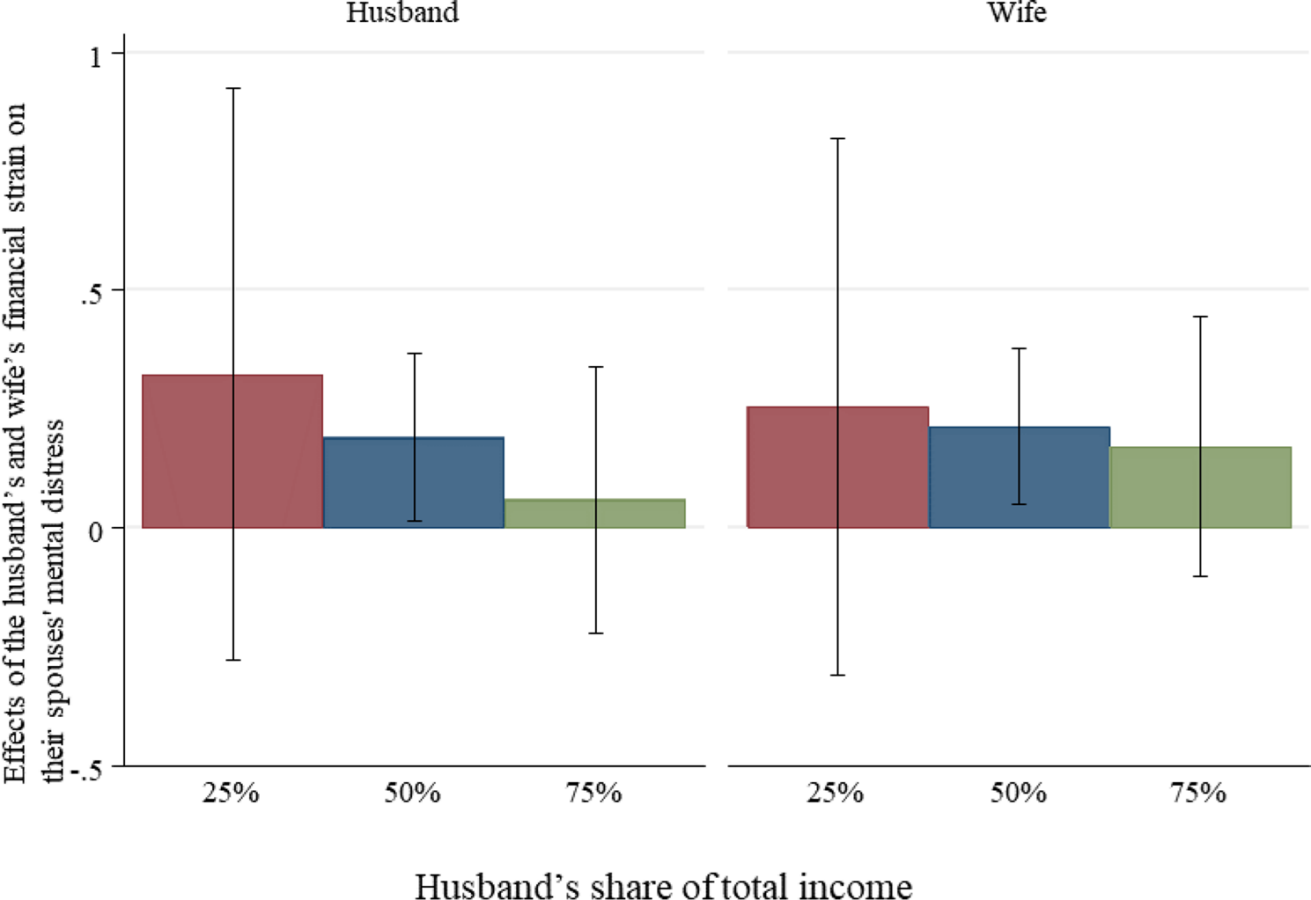
Effects of the husband’s and wife’s financial strain on their partner’s mental distress by the husband’s share of couple’s income

Figures 1 and 2 illustrate the interaction effects between financial strain and income share for the husband and wife. For the husband, the negative effect of his own financial strain on his mental distress is moderated by his income share, with higher income shares mitigating his distress. In contrast, the effect of his wife’s financial strain on his mental distress remains similar regardless of his income share. This suggests that being the primary breadwinner serves as a protective factor for the husband against his mental strain associated with his own financial difficulties. For the wife, the effects of both her own and her husband’s financial strain on her mental distress remain similar, irrespective of her husband’s income share.

Panel B investigates the role of the wife’s share of housework in moderating the effect of financial strain on mental distress. The results reveal the interaction between the husband’s financial strain and the wife’s share of housework on the husband’s mental distress is not statistically significant (B = -1.040, *p* > 0.05 for the linear term; B = 0.523, *p* > 0.05 for the quadratic term). The interaction between the wife’s financial strain and wife’s share of housework on her husband’s mental distress is also not statistically significant (B = 0.102, *p* > 0.05 for the linear term; B = -0.082, *p* > 0.05 for the quadratic term). For the wife, neither the interaction between her husband’s financial strain and her share of housework (B = 0.622, *p* > 0.05 for the linear term; B = -0.526, *p* > 0.05 for the quadratic term) nor the interaction between her own financial strain and her share of housework (B = -0.019, *p* > 0.05 for the linear term; B = 0.128, *p* > 0.05 for the quadratic term) is statistically significant, suggesting that the amount of housework performed by the wife does not significantly moderate the effect of financial strain on their mental distress. These findings support H5a and H6a.

**Fig. 3:**
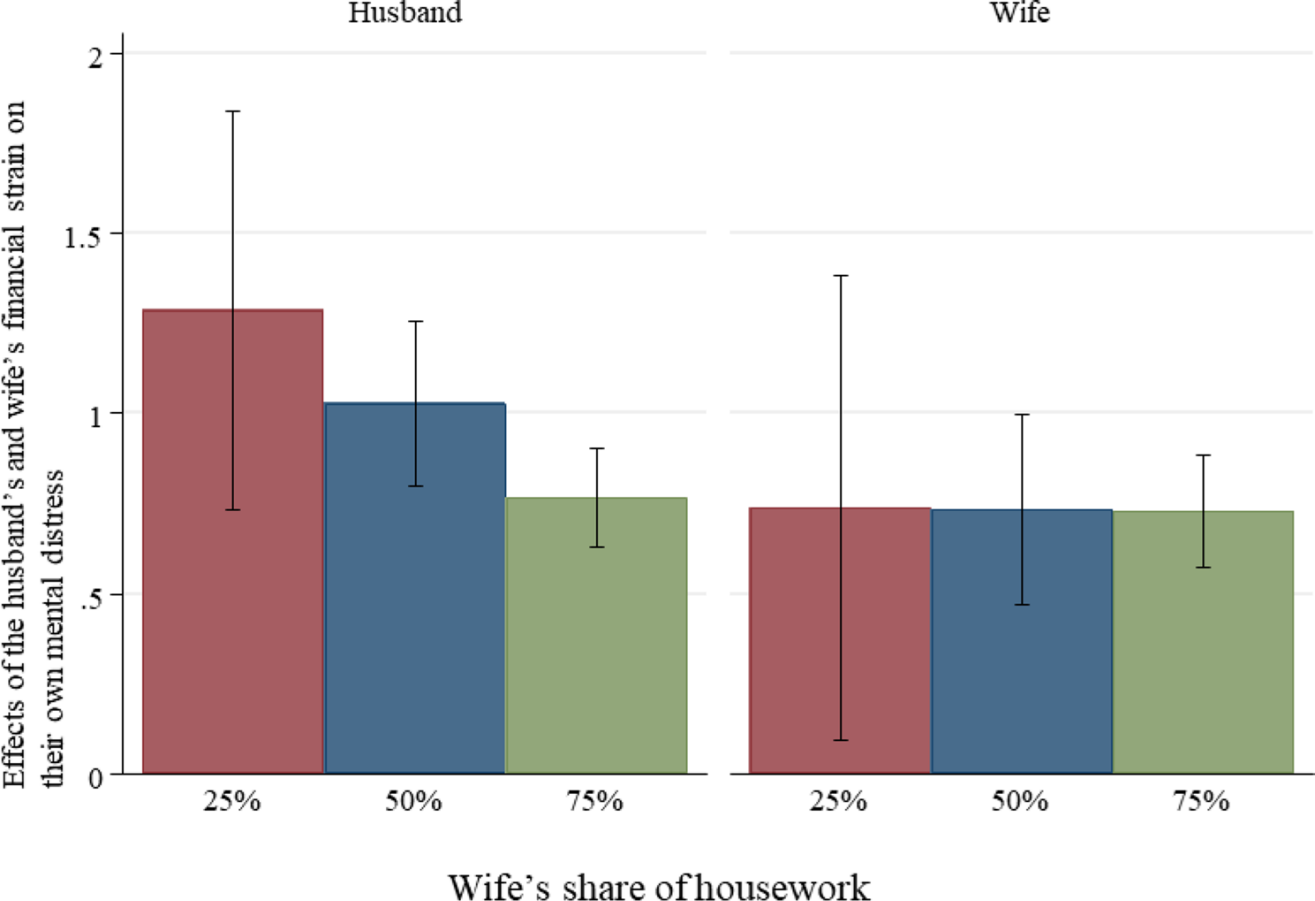
Effects of the husband’s and wife’s financial strain on their own mental distress by the wife’s share of housework

**Fig. 4:**
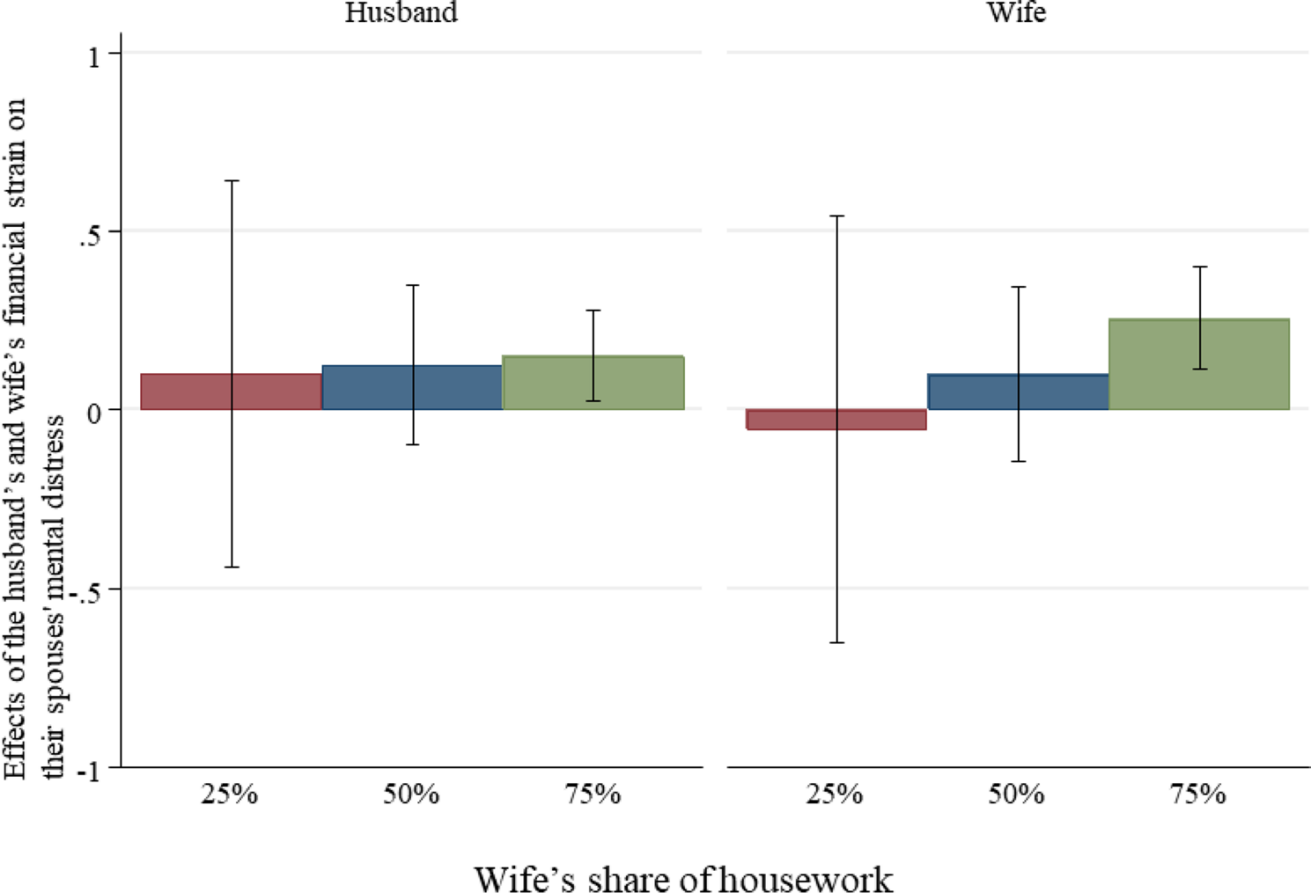
Effects of the husband’s and wife’s financial strain on their partner’s mental distress by the wife’s share of housework

Figures 3 and 4 illustrate the interaction effects, highlighting the role of housework division in moderating the effect of financial strain on mental distress for both the husband and wife. For the husband, his mental distress associated with his own financial strain slightly decreases as his wife’s share of housework increases, while his distress related to his wife’s financial strain remains consistent regardless of her share of housework. For the wife, her mental distress associated with her own financial strain remains stable across different shares of housework, but her distress linked to her husband’s financial strain slightly increases as her share of housework rises. These findings indicate that housework has a limited role in alleviating the psychological effects of financial strain, especially for the wife.

## Discussion

Our study deepens the understanding of the complex relationship between financial strain and mental distress by incorporating a dyadic perspective that accounts for the gendered division of labor within couples. By moving beyond an individualistic focus, this study demonstrates that financial strain influences not just individuals but entire relationships, influencing the mental health of both partners. These findings advocate for a more nuanced approach to mental health interventions, one that considers the intricate dynamics of gender roles and financial stress within the context of couple relationships. Such a comprehensive perspective is crucial for developing strategies that support mental health in the face of economic challenges.

The public health implications of our findings underscore the need for more comprehensive strategies. Traditional gender roles, particularly in income provision, may offer psychological protection for husbands but do not provide the same benefit for wives, indicating a need for targeted mental health interventions that address the unique stressors faced by women, especially those related to the undervaluation of domestic labor. Promoting gender equity in both income generation and domestic responsibilities could help distribute the psychological burden more evenly, potentially reducing overall mental distress. Furthermore, since housework does not significantly buffer the mental health effect of financial strain, public health policies should advocate for greater societal recognition and support for domestic labor, including mental health resources for caregivers. Finally, couple-based approaches that encourage shared financial and domestic responsibilities may be more effective in managing stress and improving mental health outcomes for both partners.

Our study has several limitations. First, our analysis focused exclusively on mental distress as the dependent variable. While mental distress provides valuable insights into psychological well-being, it represents only one aspect of overall well-being. Future research would benefit from exploring additional outcomes such as life satisfaction and subjective well-being to provide a more comprehensive understanding of how financial strain influences individuals in different-sex couples. Second, our measure of financial strain relied on a self-reported and single-dimensional question of the questionnaire, which may introduce potential inaccuracies and biases. Exploring more objective and diverse measures of financial strain could improve the reliability of the findings. For instance, incorporating indicators such as household debt situations, whether has access to financial resources, and the ability to pay rent or mortgage on time would offer a more nuanced assessment. Third, our analysis employed fixed effect models, which are unable to account for time-varying variables. This constraint may overlook important dynamic factors that could influence the relationship between financial strain and mental distress over time. Fouth, our measurement of housework does not include childcare, a significant component of domestic labor for families with young children, nor does it conclude the cognitive load associated with housework. Non-routine housework may also not be adequately captured by the questionnaire. Future studies could incorporate childcare responsibilities, cognitive load, and non-routine tasks to achieve a more comprehensive understanding of domestic labor dynamics and their effects. Finally, our study used nationally representative data from the UK, which may limit the generalizability of our findings to other cultural and economic contexts. To address this, future research may consider cross-country studies to examine whether the observed patterns hold in different countries with varying economic conditions and cultural norms. This would provide a more robust understanding of the spousal crossover effects of financial strain and the role of gendered divisions of labor across diverse settings.

## Conclusion

Using nationally representative longitudinal dyadic data from the UK and fixed effect models, this study investigates the interplay between financial strain and mental distress among husbands and wives, with a focus on the different relationship by gendered division of labor in income and housework. Our findings offer several important insights.

First, we find that for both the husband and wife, financial strain is not only associated with their own mental distress but also their spouses’ mental distress, although the spousal cross-over effects have smaller effect sizes. It extends the conversation beyond individual experiences to underscore the interconnected nature of dyadic relationships [59, 60]. This insight has profound implications for both research and intervention strategies. It emphasizes the need for a holistic approach in understanding and addressing mental health issues, recognizing that individual well-being is intricately tied to the well-being of one’s partner [61]. This suggests that mental health interventions should not only target individuals but also consider the relational context within which individuals operate. Couples facing financial strain might benefit more from interventions that address their shared experiences and coping strategies rather than focusing on individuals in isolation.

Second, our findings reveal that the husband’s mental distress associated with his own financial strain decreases as he assumes a larger share of the couple’s income. However, this protective effect does not extend to alleviating the wife’s mental distress associated with her own or her husband’s financial strain. This highlights the profound influence of the traditional gender norms on mental health, suggesting that fulfilling conventional expectations—where the husband is the primary breadwinner—can serve as a protective factor for the husband’s mental distress from his own financial challenges. This underscores the deep-seated nature of gender roles in division of income in shaping individuals’ psychological responses to financial pressures, emphasizing the resilience of traditional gender norms and their ongoing effect on mental health within modern marital relationships [10]. Furthermore, the protective effect of adhering to traditional roles suggests a need for broader societal support systems that can buffer against financial strain without relying on rigid gender norms.

Third, we observe that the wife’s share of housework does not modify the effect of financial strain on her husband’s or her own mental distress. This suggests that traditional gender roles in division of labor, where the wife takes on greater housework responsibilities, may not provide the anticipated protective benefits against financial strain. The benefits of adhering to traditional gender roles do not appear to be reciprocal in handling financial stress, indicating a need to reassess the effectiveness of these roles in modern contexts [50, 62]. Policies and interventions aimed at reducing financial strain should focus on addressing the root causes of financial precarity rather than relying on traditional family structures to mitigate its effects. It may also suggest that policies and interventions aimed at reducing financial strain should focus on addressing the root causes of financial precarity rather than relying on traditional family structures to buffer against its effects. One possible explanation for the differing effects of income division compared to labor division may lie in the distinct psychological and emotional dynamics associated with these roles. Traditional work roles often confer a sense of stability and protection, reinforcing partners’ expectations and support systems during financial hardships. In contrast, housework responsibilities may not carry the same perceived value or emotional support, potentially resulting in feelings of resentment or inequity [38, 63].

## Data Availability

Data available at https://ukdataservice.ac.uk/.

## Footnotes

1 In this context, husbands and wives refer to men and women in both married and unmarried co-residing couples.

2 In this context, the term “effect” refers to a statistical effect and does not imply a causal effect. The same applies throughout the rest of the text.

## Abbreviations

UK: United Kingdom
UKHLS: United Kingdom Household Longitudinal Study
GHQ: General Health Questionnaire
M: Means
SD: Standard deviations
